# First-trimester choroid plexus-to-head ratio: a novel sonographic marker for the early detection of fetal central nervous system malformations

**DOI:** 10.3389/fmed.2025.1637954

**Published:** 2025-11-05

**Authors:** Xianghua Xue, Peng Tu, Xiaohang Zhang, Suzhen Ran, Xuemei Zhang

**Affiliations:** ^1^Department of Ultrasound, Women and Children’s Hospital of Chongqing Medical University, Chongqing, China; ^2^Department of Ultrasound, Chongqing Health Center for Women and Children, Chongqing, China

**Keywords:** prenatal diagnosis, choroid plexus, head, nervous system malformations, first trimester

## Abstract

**Background:**

Prenatal diagnosis of nervous system malformations associated with the abnormal development of cerebrospinal fluid is most commonly made in the mid-trimester. During early gestation, the choroid plexus is the dominant structure occupying the lateral ventricles. Detection of abnormally positioned choroid plexuses within the fetal head may serve as a novel sonographic marker for the early identification of these malformations.

**Methods:**

This was a retrospective study of singleton pregnancies with nervous system abnormalities. In the first trimester, the choroid plexus area (CPA), choroid plexus length (CPL), occipitofrontal diameter (OFD), head area (HA), head circumference (HC), abdominal circumference (AC), and the distance between the anterior margin of the choroid plexus (CP) and the medial side of the frontal bone (CFD) were measured in the transventricular axial plane. The ratios of CPA/HA, CPL/OFD, CFD/OFD, and HC/AC were also assessed.

**Results:**

A total of 256 fetuses were included in the control group, and a reference range was established. In total, 52 cases with nervous system abnormalities associated with abnormal cerebrospinal fluid development were confirmed. In the control group, the ratios of CPA/HA and CPL/OFD decreased (*r* = −0.499, −0.216, respectively, both *p* < 0.001), while the CFD/OFD ratio increased (*r* = 0.445, *p* < 0.001). Compared with the control group, scatter diagrams and box-and-whiskers plots showed ventriculomegaly (VM) fetuses had significantly decreased CPA/HA and CPL/OFD ratios (*p* < 0.001), but increased CFD/OFD and HC/AC ratios (*p* < 0.001). The CPA/HA, CPL/OFD, and CFD/OFD ratios were all significantly higher in fetuses with open spina bifida (OSB) (*p* < 0.05). Fetuses with microcephaly showed a significantly higher CPA/HA ratio (*p* < 0.001), higher CPL/OFD ratio (*p* < 0.001), and lower HC/AC ratio (*p* < 0.05).

**Conclusion:**

Choroid-plexus-to-head ratios offer an objective means of assessing nervous system malformations associated with abnormal cerebrospinal fluid development during the first trimester.

## Background

To identify early fetal brain abnormalities, such as alobar holoprosencephaly and open brain deformity, the integrity of the fetal cranium and falx cerebri is examined using transventricular axial plane scanning at 11–13 weeks’ gestation. In addition, the choroid plexus (CP) and lateral ventricles are among the most easily observed structures during the period (1–3). The CPs play an important role in the proliferation of neural stem cells and the regulation of brain development (4). Prenatal diagnosis of nervous system malformations associated with abnormal development of cerebrospinal fluid is most commonly made in the mid-trimester (5, 6). The assessment of choroid-plexus-to-head ratios in the first trimester may provide new insights into the development of these diseases. However, during early and middle pregnancy, the CPs predominantly occupy the lateral ventricles. Moreover, the diagnostic criteria for nervous system abnormalities associated with abnormal cerebrospinal fluid development, such as ventriculomegaly (VM), open spina bifida (OSB), and microcephaly in the first trimester, vary considerably.

The identification of contracted CPs may prompt suspicion of ventricular dilatation and trigger further evaluation of other defects associated with aneuploidies (7, 8). As correctly noted, VM in the majority of cases represents an indirect sign of other central nervous system malformations or pathological conditions, whereas isolated VM is not considered a true malformation. First-trimester prediction of VM using choroid plexus to lateral ventricle ratios appears promising (9). Considering the time for genetic examination and prenatal management, it is imperative to investigate independent indicators in the first trimester for nervous system malformations associated with abnormal cerebrospinal fluid development.

This study had two main objectives: first, to establish normal distributions for a range of choroid-plexus-to-head measurements; and second, to evaluate these measurements in different types of nervous system malformations associated with abnormal cerebrospinal fluid development and compare them with the normal group.

## Methods

### Study participants

This retrospective study focused on singleton pregnancies with nervous system abnormalities associated with abnormal cerebrospinal fluid development after 16 weeks’ gestation in our center, spanning from June 2017 to June 2023, using first-trimester ultrasound imaging. A total of 108,941 fetuses underwent routine first-trimester ultrasound examination in the center during 11–14 weeks’ gestation. All cases were determined through a comprehensive search of the medical information database. Our center is an academic tertiary referral medical unit, where routine first-trimester screening is also offered to low-risk pregnant women.

Inclusion criteria for the normal group: (1) fetal crown-rump length (CRL) range: 45–84 mm; (2) low-risk pregnancy, with no history of anemia, hypertension, heart disease, smoking, or drinking; (3) normal neurological development assessment of the newborn within 1 year after birth. Exclusion criteria included: (1) incomplete prenatal data and lack of follow-up results; (2) severe brain malformations, such as alobar holoprosencephaly, Dandy–Walker malformation, or agenesis of the corpus callosum; and (3) fetuses with other structural or genetic syndromes.

Nervous system abnormalities associated with abnormal cerebrospinal fluid development, including isolated VM, OSB, and microcephaly, were enrolled in the abnormal group. VM was further classified as mild when the atrial width was 10–15 mm and severe when it was ≥15 mm (2, 10). Microcephaly was defined as mild when the head circumference was below 2–3 standard deviations (SD) from the mean, and severe when it was more than 3 SD below the mean (11, 12). All abnormal fetuses underwent at least one ultrasound scan in the early stage, with complete follow-up data available for mid- and late pregnancy. Exclusion criteria included: (1) unclear image quality due to maternal obesity or unfavorable fetal position, (2) VM secondary to intracranial hemorrhage or infection in mid- or late pregnancy, and (3) fetuses with other intracranial structural malformations. All included fetuses were confirmed either prenatally or postnatally (including after the termination of pregnancy) by magnetic resonance imaging (MRI) or autopsy.

Gestational age was determined based on the last menstrual period and confirmed by the CRL measurement. As this study involved a retrospective review of patient data, ethical approval was not needed, and all pregnant women provided informed consent for early pregnancy ultrasound examination.

### Ultrasound examinations

**Figure 1:**
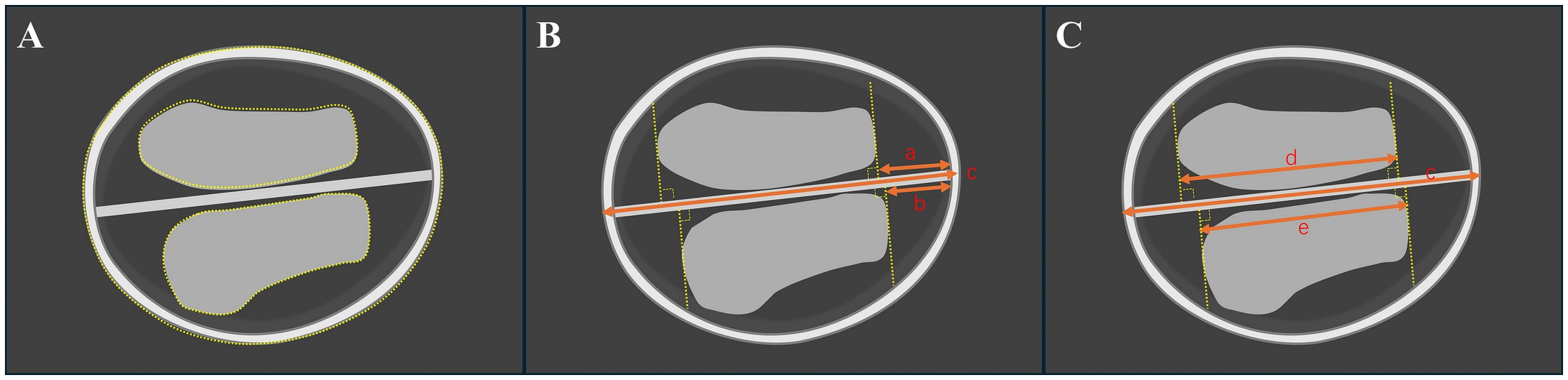
Illustration of the standard axial butterfly plane in the first trimester of the brain in a normal fetus. **(A)** Measurement of the choroid plexus and head areas by a dashed line. **(B)** Measurement of the occipitofrontal diameter (OFD) and the vertical distance from the apex of the CP to the medial side of the frontal bone (CFD). **(C)** Measurement of choroid plexus length (CPL) and CFD. The “a and b” mean CFD. The “c” means OFD. The “d and e” mean CPL.

Ultrasound measurements were conducted using the Voluson E8/E10 system (GE Medical Systems, Zipf, Austria) or Samsung WS80A (Samsung Medison, Seoul, South Korea). Nuchal translucency (NT) and CRL (measured in mm) values were measured in the mid-sagittal plane. The standard measurement in the transthalamic plane has been reported (2): (1) Ultrasonic beam scanned through the temporal window, which means the falx cerebri was nearly perpendicular to the acoustic beam. (2) The integrity of bony contours, a separate choroid plexus (butterfly sign), and falx cerebri were clearly presented. (3) Images were enlarged to 1/2–1/3 of the screen (Figure 1A).

**Figure 2:**
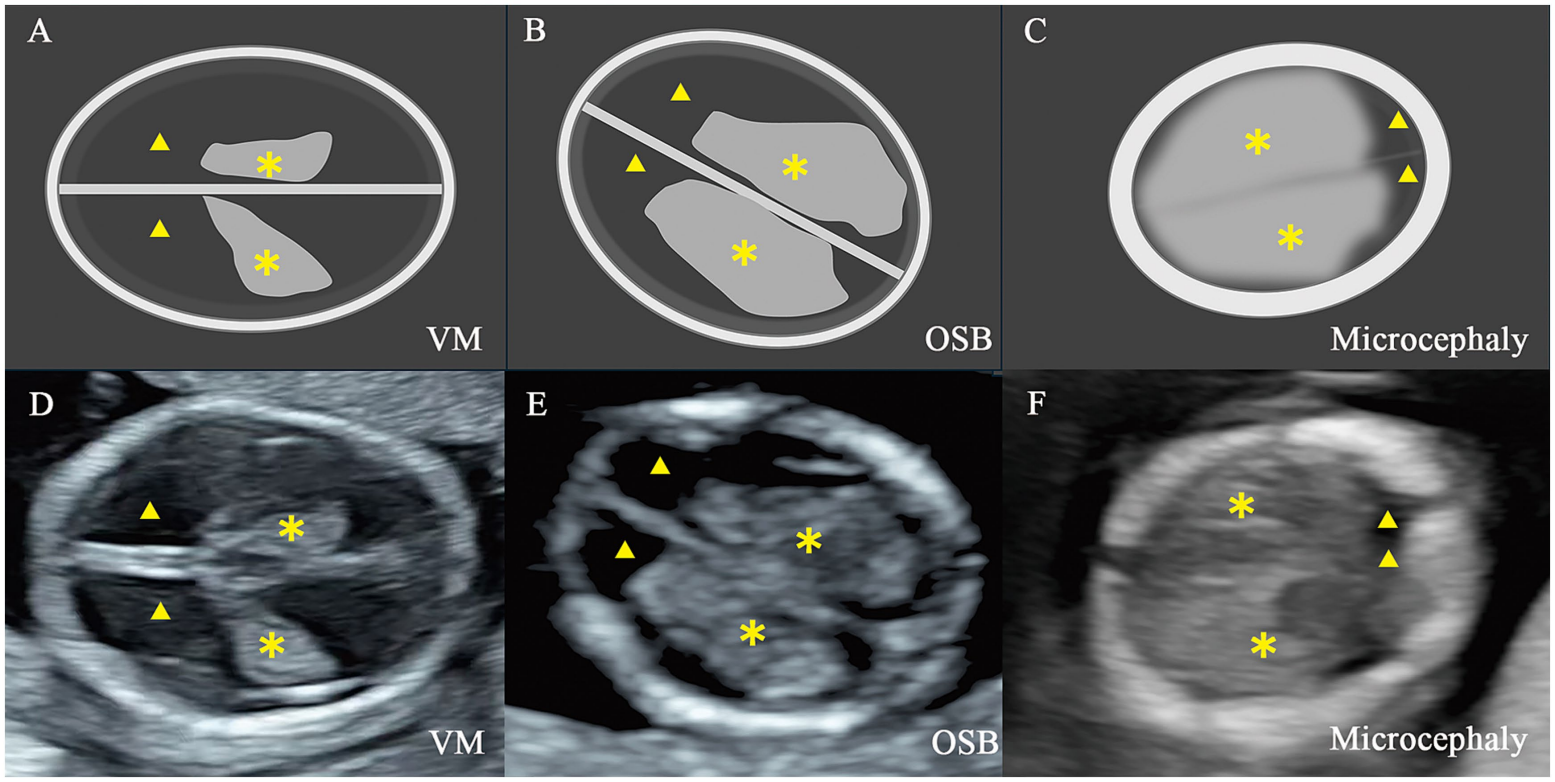
Illustration and two-dimensional view of the standard axial butterfly plane in fetus with nervous system abnormalities. **(A,D)** Fetus with ventriculomegaly (VM). **(B,E)** Fetus with open spina bifida (OSB). **(C,F)** Fetus with microcephaly. ▲, the lateral ventricles; ✽, the choroid plexus.

For the choroid plexus area (CPA), head area (HA), head circumference (HC), and abdominal circumference (AC) are measured using the tracing method along their perimeter. Two lines are drawn separately through the apex of the CP perpendicular to the falx cerebri. The distance between the two lines is defined as the choroid plexus length (CPL), and the vertical distance from the apex of the CP to the medial side of the frontal bone is defined as CFD. The “outer-to-outer” method was used to measure the CPL and occipitofrontal diameter (OFD), the “outer-to-inner” method was used to measure the CFD, which were both at the level of the choroid plexus (Figures 1, 2). Importantly, in order to minimize errors caused by the inconsistent morphology of the choroid plexus on both sides, the CPL = (left choroid plexus length + right choroid plexus length)/2 and the CPA = (left choroid plexus area + right choroid plexus area). The ratios of choroid-plexus-to-head were assessed, including the ratios of CPA/HA, CPL/OFD, CFD/OFD, and HC/AC.

All ultrasound examinations in the first trimester and the diagnosis for abnormal cases were made by two experienced physicians (X.H.Z. and X.M.Z.). All parameters were measured offline by two researchers (X.X.H. and P.T.), blinded to the clinical diagnosis, and an average of two measurements was taken.

### Statistical analysis

Data were analyzed by the software SPSS Statistical for Windows version 17.0 and GraphPad Prism 4. A *p-*value of < 0.05 was considered statistically significant. The normality of continuous variables was assessed using the Kolmogorov–Smirnov test. Data were expressed as mean ± standard deviation (SD) for normally distributed variables or as median (interquartile range, IQR) for non-normally distributed variables. Meanwhile, categorical variables were presented as numbers and percentages. In 20 normal fetuses, repeatability was assessed by calculating the intraclass correlation coefficients (ICCs) for intra- and interobserver reliability and utilizing the Bland–Altman method at different gestational ages. Pearson’s correlation analysis was used to demonstrate the correlation between each parameter and CRL.

The normal and abnormal groups were screened in an approximate 5:1 ratio. Simple random sampling was applied to ensure comparability between groups. A 90% prediction interval (5th, 50th, and 95th percentiles) around the regression line was estimated based on the relationship to CRL. Box-and-whisker plots were also constructed according to the type of pathology. Comparison of quantitative variables was performed using the Kruskal–Wallis test. Receiver operating characteristic (ROC) curve analysis was conducted to evaluate predictive performance, and variables with an area under the curve (AUC) < 0.5 were excluded from subsequent analyses.

## Results

### Normal fetuses and reference ranges

**Figure 3:**
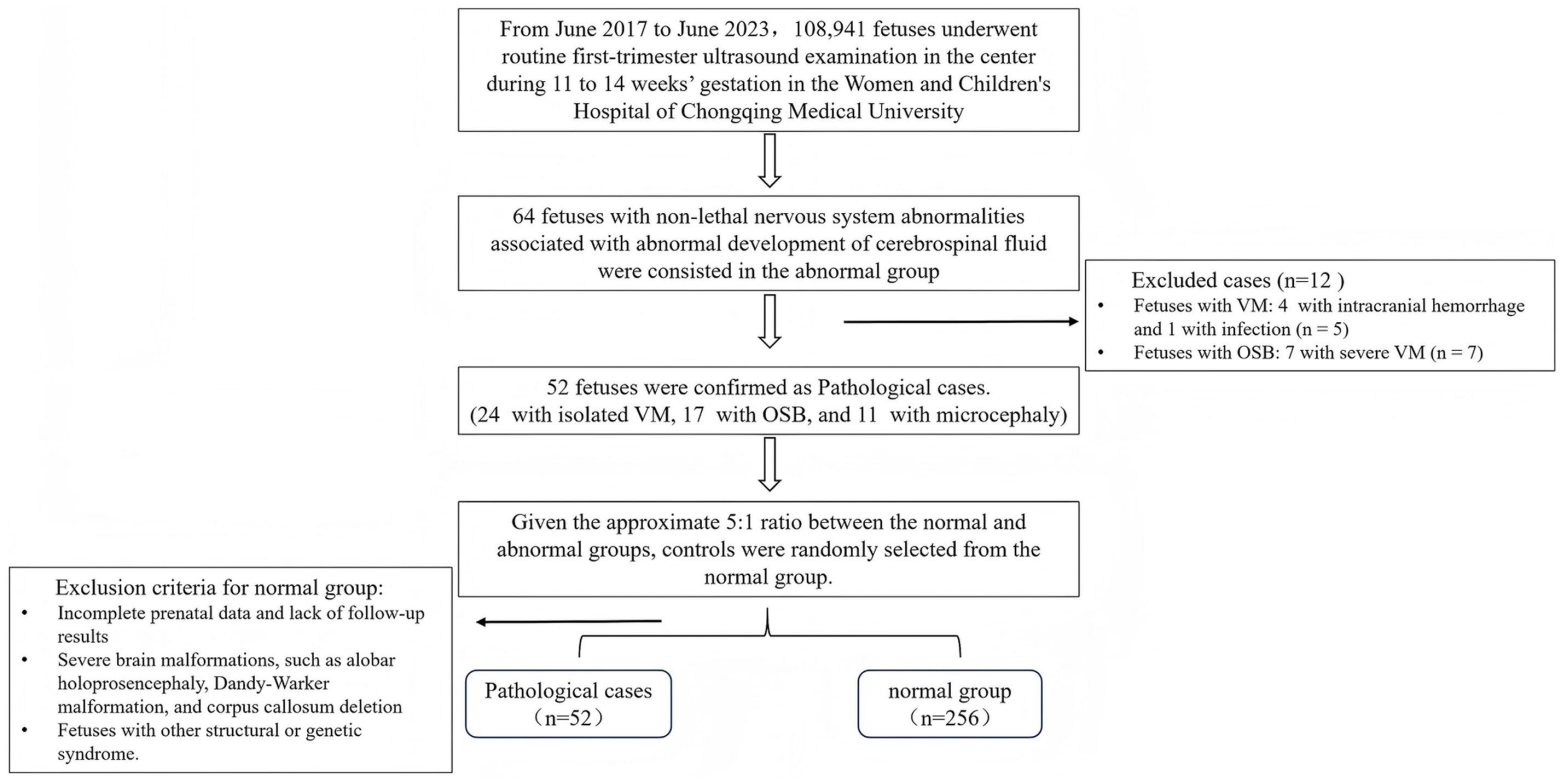
Flowchart for patient selection. VM, ventriculomegaly; OSB, open spina bifida.

A total of 256 singleton normal fetuses were included in the control group analysis (Figure 3), and a reference range was established for each parameter. There was good intra- and inter-observer reliability (ICC > 75%) for all measurements (Table 1). Bland–Altman plots are presented in Supplementary Figures S1, S2. The difference in pregnancy characteristics and parameters is summarized in Table 2. As indicated, there was no significant difference between the control group and abnormal groups with respect to maternal age (*p* = 0.29) and CRL (*p* = 0.97). However, the NT was found significantly lower in the control group (*p* < 0.05).

**Table 1:** Intra-and interobserver reliability of measurements for parameters in choroid-plexus-to-head section in first trimester.

Measurement	ICC (95% CI)	
	Intraobserver	Interobserver
CPA/HA ratio	0.74 (0.45–0.89)	0.80 (0.52–0.92)
CPL/OFD ratio	0.92 (0.57–0.97)	0.92 (0.80–0.97)
CFD/OFD ratio	0.92 (0.81–0.97)	0.91 (0.79–0.97)
HC/AC ratio	0.79 (0.56–0.91)	0.74 (0.46–0.89)

CPA, choroid plexus area; HA, head area; CPL, choroid plexus length; OFD, occipitofrontal diameter; CFD, the vertical distance from the apex of the CP to the medial side of the frontal bone; HC, head circumference; AC, abdominal circumference.

**Table 2:** Baseline characteristics and ultrasound measurements in cases with nervous system abnormalities compared with controls.

Variable	Normal (*n* = 256)	VM (*n* = 24)	OSB (*n* = 17)	Microcephaly (*n* = 11)	*p*-value
Maternal age (years)	29.0 ± 3.9	29.5 ± 4.8	29.5 ± 4.5	31.2 ± 3.9	0.29
CRL (mm)	63.8 ± 9.2	63.0 ± 8.0	63.2 ± 8.2	63.7 ± 6.7	0.97
Nuchal translucency (mm)	1.35 (1.10–1.70)	1.45 (1.30–2.00)	1.50 (1.35–1.85)	1.50 (1.20–1.60)	<0.05
CPA/HA ratio	0.31 (0.28–0.34)	0.27 (0.20–0.31)	0.37 (0.32–0.42)	0.51 (0.44–0.54)	<0.001
CPL/OFD ratio	0.54 (0.51–0.57)	0.47 (0.40–0.52)	0.60 (0.55–0.64)	0.63 (0.59–0.67)	<0.001
CFD/OFD ratio	0.09 (0.06–0.11)	0.23 (0.19–0.25)	0.15 (0.08–0.20)	0.07 (0.05–0.09)	<0.001
HC/AC ratio	1.17 (1.12–1.23)	1.33 (1.21–1.45)	1.16 (1.07–1.22)	0.96 (0.91–1.10)	<0.001

CPA, choroid plexus area; HA, head area; CPL, choroid plexus length; OFD, occipitofrontal diameter; CFD, the vertical distance from the apex of the CP to the medial side of the frontal bone; HC, head circumference; AC, abdominal circumference.

**Figure 4:**
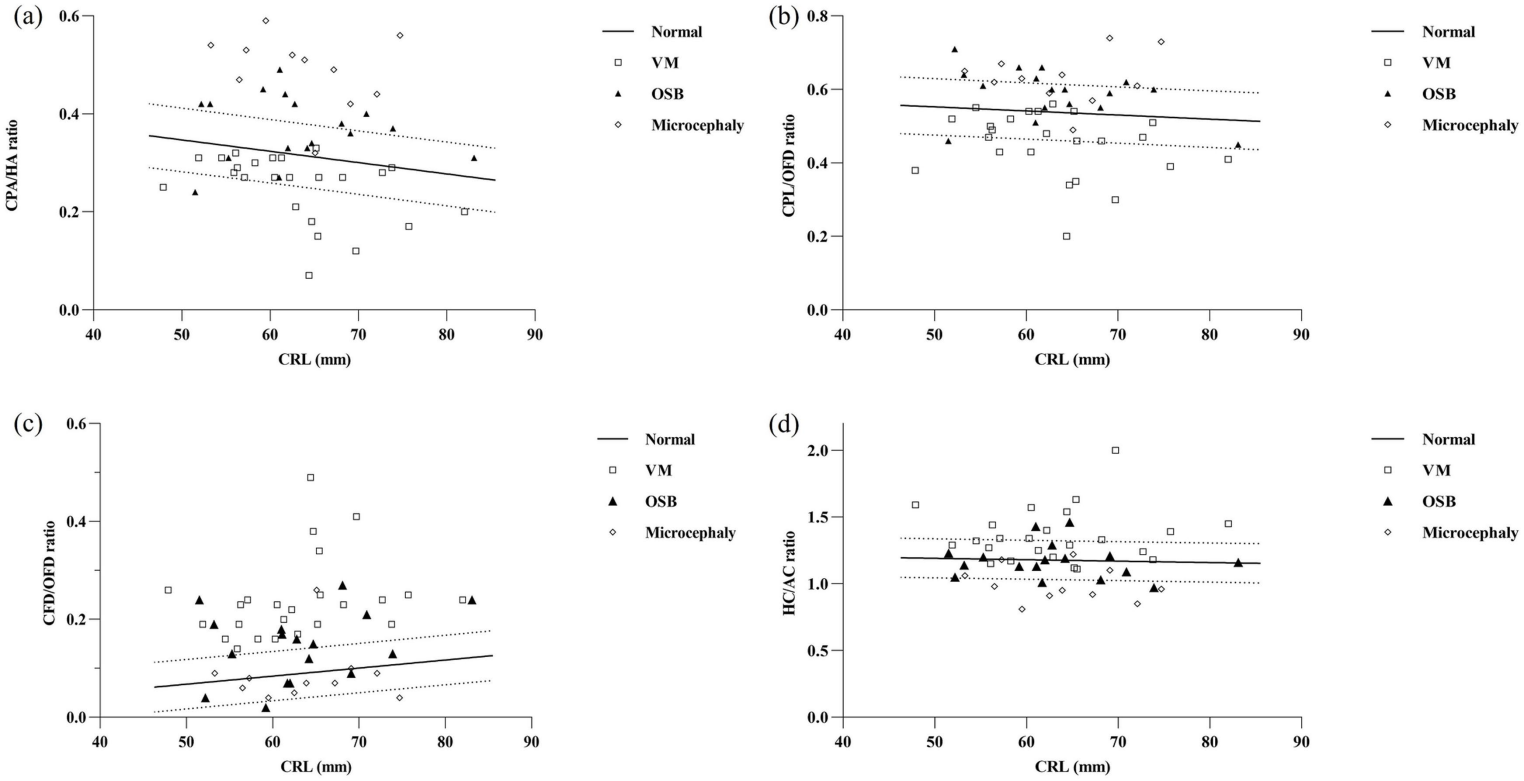
Parameters of the choroid-plexus-to-head section in relation to fetal crown-rump length (CRL) in the reference group and in fetuses with nervous system abnormalities. Fetuses with ventriculomegaly (*n* = 24, clear square), open spina bifida (*n* = 17, dark triangle), and microcephaly (*n* = 11, clear rhomboid) plotted against the calculated reference range (5th, 50th, and 95th percentiles). CPA, choroid plexus area; HA, head area; CPL, choroid plexus length; OFD, occipitofrontal diameter; CFD, the vertical distance from the apex of the CP to the medial side of the frontal bone; HC, head circumference; AC, abdominal circumference; VM, ventriculomegaly; OSB, open spina bifida.

Reference ranges based on CRL for CPA/HA, CPL/OFD, CFD/OFD, and HC/AC were constructed. Bivariate correlation analysis revealed no significant correlation between HC/AC and CRL (*r* = 0.09, *p* = 0.142). The ratios of CPA/HA and CPL/OFD decreased with fetal CRL, CPA/HA ratio = −0.002315 × CRL + 0.4627 (*r* = −0.499, *p* < 0.001); CPL/OFD ratio = −0.001112 × CRL + 0.6081 (*r* = −0.216, *p* < 0.001). However, the CFD/OFD ratio increased with fetal CRL, CFD/OFD ratio = 0.001646 × CRL − 0.01485 (*r* = 0.445, *p* < 0.001) (Figure 4).

### Pathological cases

**Figure 5:**
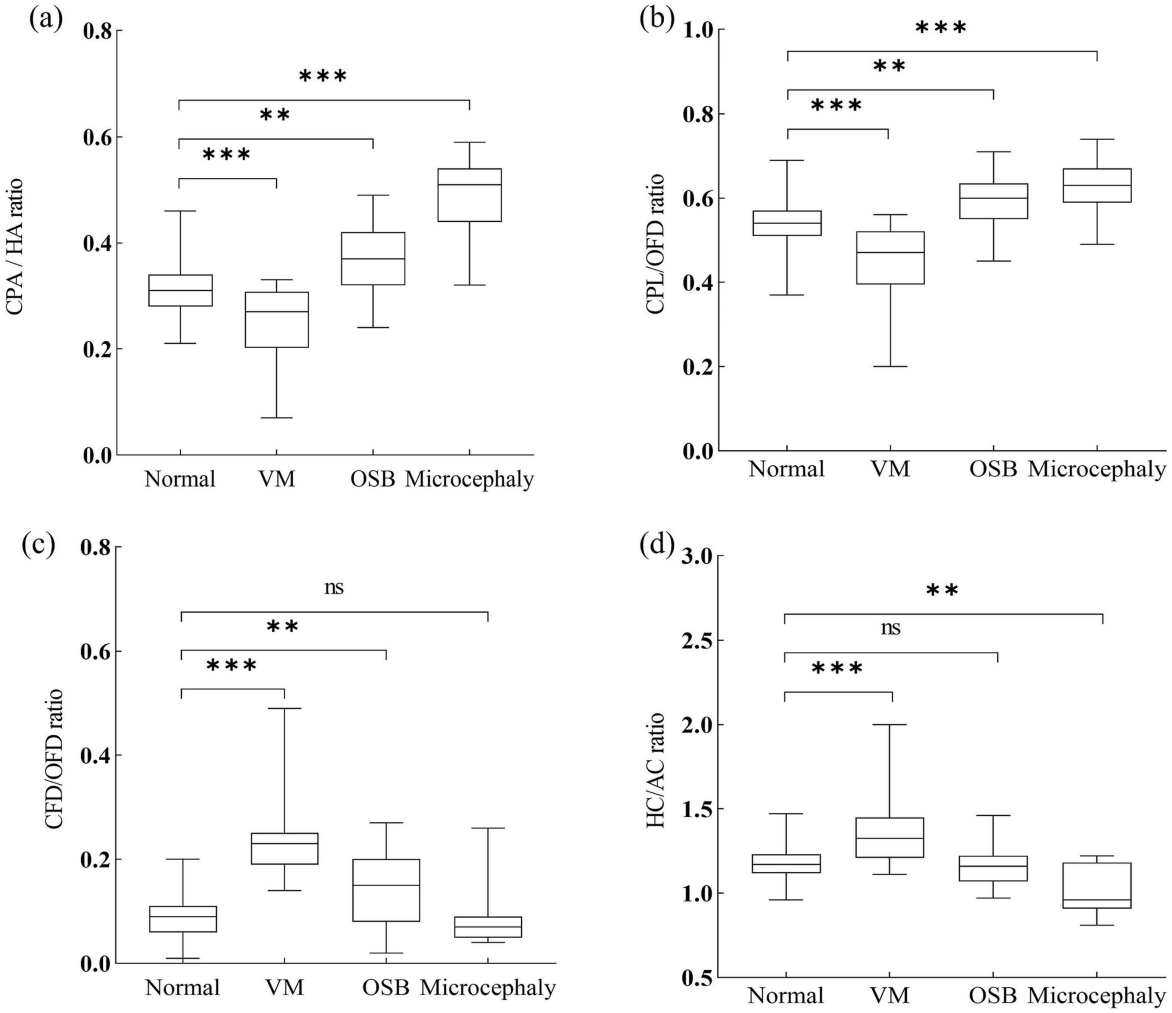
Ratios of choroid plexus to head of fetuses with nervous system abnormalities compared with controls. CPA, choroid plexus area; HA, head area; CPL, choroid plexus length; OFD, occipitofrontal diameter; CFD, the vertical distance from the apex of the CP to the medial side of the frontal bone; HC, head circumference; AC, abdominal circumference; VM, ventriculomegaly; OSB, open spina bifida. Statistical significance was performed using the Kruskal–Wallis test. ns, no significance, **p < 0.01, ***p < 0.001.

In total, 64 fetuses with nervous system abnormalities associated with the abnormal development of cerebrospinal fluid were included in the abnormal group. Five fetuses with VM were excluded during the follow-up period, including four cases with intracranial hemorrhage and one case with infection. Additionally, seven cases with OSB were detected, and severe VM cases were also excluded. Finally, 52 cases were confirmed, including 24 fetuses with isolated VM, 17 fetuses with OSB, and 11 fetuses with microcephaly. In order to present the individual values in pathological cases, we compared associated parameters with normal reference ranges (Figure 4) and median (IQR) values of variables (Figure 5).

A total of 8/24 (33.3%) cases in the fetuses with VM had CPA/HA ratio < 5th centile, 9/24 (37.5%) cases had CPL/OFD ratio < 5th centile, all 24/24 (100%) cases had CFD/OFD ratio > 95th centile, and 9/24 (37.5%) cases had HC/AC ratio > 95th centile. When compared with the control group, fetuses with VM had a significantly decreased CPA/HA and CPL/OFD ratio (*p* < 0.001), an increased CFD/OFD and HC/AC ratio (*p* < 0.001).

The proportion of CPA/HA ratio, CPL/OFD ratio, and CFD/OFD ratio above the 95th centile was 52.9% (9/17), 35.3% (6/17), and 52.9% (9/17), respectively, in fetuses with OSB. A total of 4/17 (23.5%) fetuses had abnormalities in the HC/AC ratio, including two cases with an HC/AC ratio > 95th centile and two cases with an HC/AC ratio < 5th centile. Both CPA/HA, CPL/OFD, and CFD/OFD ratios were significantly higher in the OSB group when compared with normal fetuses (*p* < 0.05); meanwhile, the HC/AC ratio showed no significant difference between fetuses with OSB and unaffected fetuses (*p* > 0.05).

**Figure 6:**
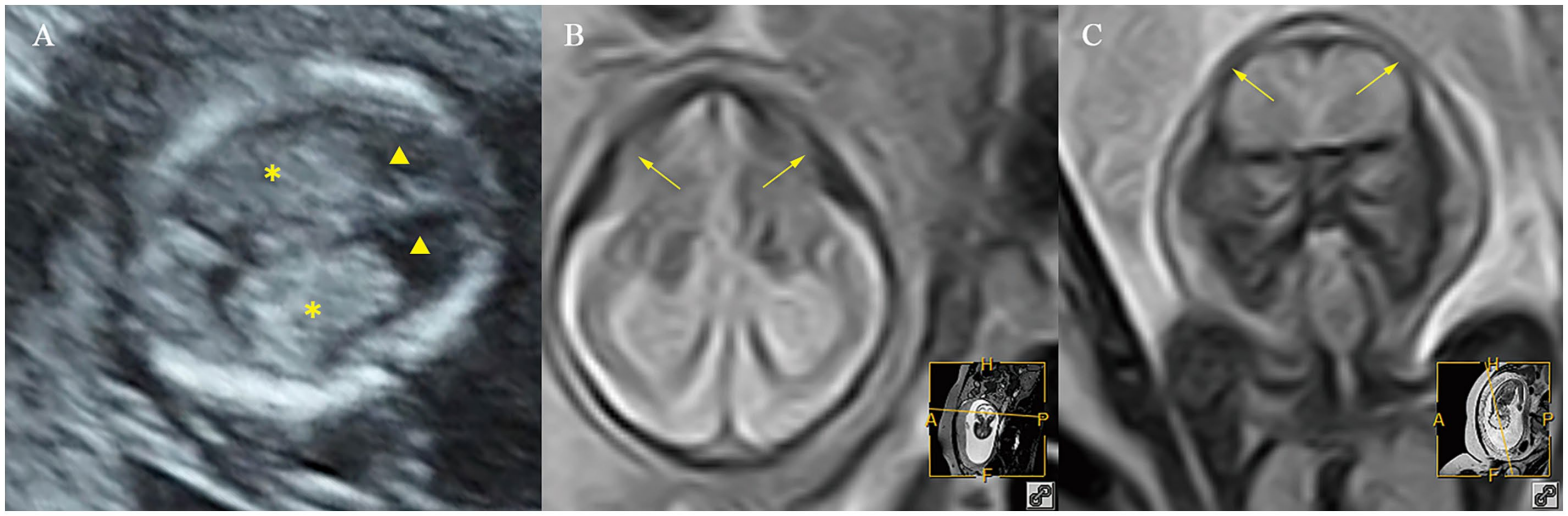
Ultrasound and MR images of a fetus with microcephaly at early and mid-gestation. **(A)** Ultrasound images at early gestation. **(B,C)** MR images at mid-gestation. ▲, the lateral ventricles; ✽, the choroid plexus; ⟶, abnormal cranial morphology.

A total of 10/11 (90.9%) cases in the fetuses with microcephaly had CPA/HA ratio > 95th centile, 6/11 (54.5%) cases had CPL/OFD ratio > 95th centile, and 7/11 (63.6%) cases had HC/AC ratio < 5th centile. A total of 2/11 (18.2%) cases had abnormalities in the CFD/OFD ratio, including one case with a CFD/OFD ratio > 95th centile and one case with a CFD/OFD ratio < 5th. Fetuses with microcephaly showed a significantly higher CPA/HA ratio (*p* < 0.001), higher CPL/OFD ratio (*p* < 0.001), and lower HC/AC ratio (*p* < 0.05); the difference in CFD/OFD ratio did not reach statistical significance (*p* > 0.05) (Figure 6).

**Figure 7:**
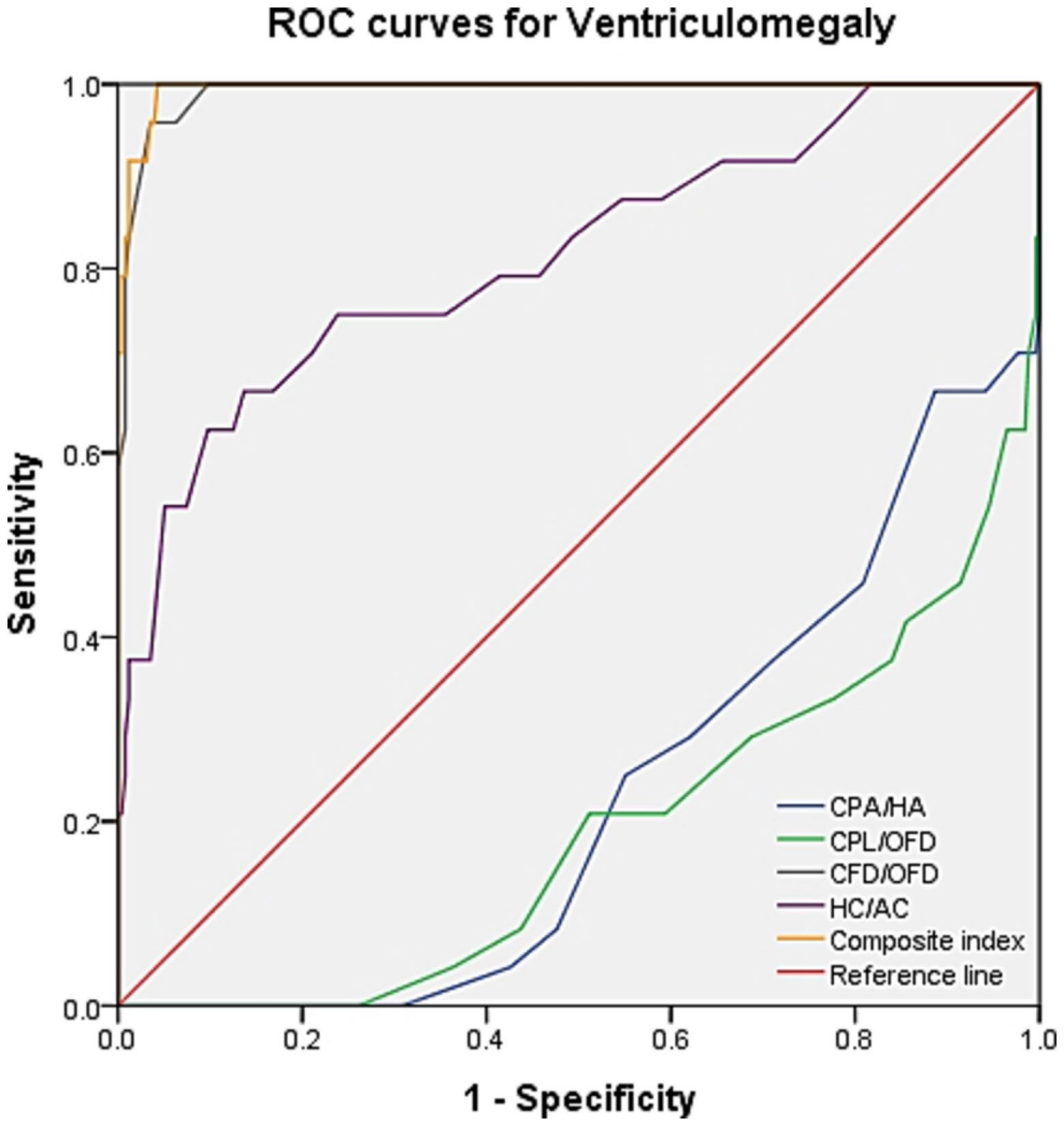
ROC curves for ventriculomegaly. CPA, choroid plexus area; HA, head area; CPL, choroid plexus length; OFD, occipitofrontal diameter; CFD, the vertical distance from the apex of the CP to the medial side of the frontal bone; HC, head circumference; AC, abdominal circumference.

**Figure 8:**
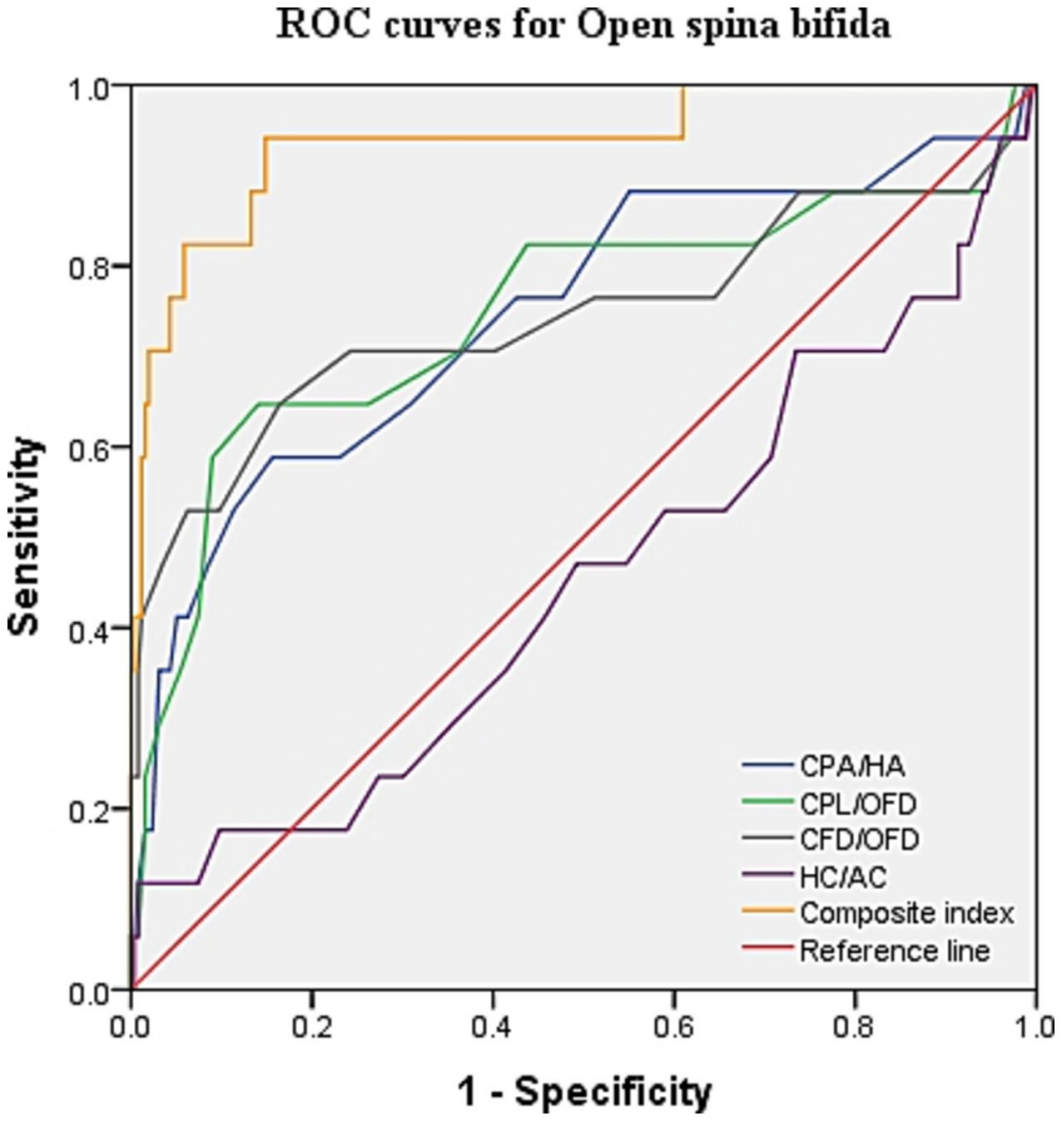
ROC curves for open spina bifida. CPA, choroid plexus area; HA, head area; CPL, choroid plexus length; OFD, occipitofrontal diameter; CFD, the vertical distance from the apex of the CP to the medial side of the frontal bone; HC, head circumference; AC, abdominal circumference.

**Figure 9:**
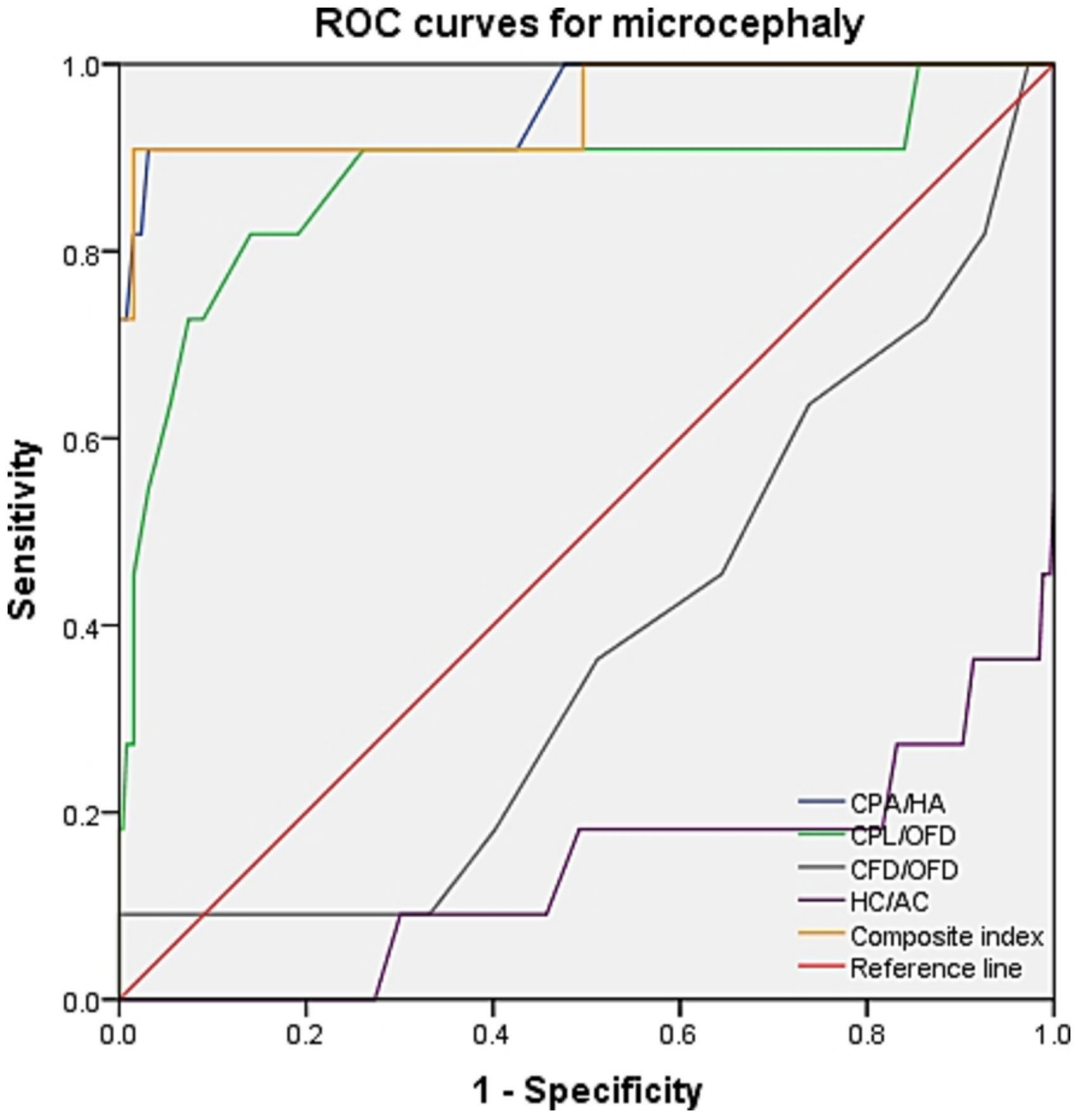
ROC curves for microcephaly. CPA, choroid plexus area; HA, head area; CPL, choroid plexus length; OFD, occipitofrontal diameter; CFD, the vertical distance from the apex of the CP to the medial side of the frontal bone; HC, head circumference; AC, abdominal circumference.

ROC curve analysis was performed to evaluate the diagnostic performance within each anomaly group (Figures 7–9). The combined index yielded a higher AUC than the individual parameters, indicating enhanced predictive efficiency. The AUC values were 0.995 (95% CI: 0.990–1.000), 0.937 (95% CI: 0.868–1.000), and 0.952 (95% CI: 0.868–1.000) across the respective anomaly groups (Table 3).

**Table 3:** Results of ROC curve analysis for individual and composite index.

Disease categories	Variable	AUC (95%CI)	Youden’s index	Sensitivity	Specificity
Ventriculomegaly	CPA/HA	0.229 (0.141–0.317)	−0.004	0	0.996
	CPL/OFD	0.191 (0.098–0.284)	−0.004	0	0.996
	CFD/OFD	0.992 (0.983–1.000)	0.923	0.958	0.965
	HC/AC	0.813 (0.710–0.917)	0.53	0.667	0.863
	Composite index	0.995 (0.990–1.000)	0.957	1.0	0.957
Open spina bifida	CPA/HA	0.746 (0.603–0.889)	0.432	0.588	0.844
	CPL/OFD	0.747 (0.592–0.902)	0.506	0.647	0.859
	CFD/OFD	0.745 (0.580–0.909)	0.483	0.647	0.836
	HC/AC	0.452 (0.295–0.610)	0.11	0.118	0.992
	Composite index	0.937 (0.868–1.000)	0.793	0.941	0.852
Microcephaly	CPA/HA	0.955 (0.879–1.000)	0.878	0.909	0.969
	CPL/OFD	0.877 (0.735–1.000)	0.677	0.818	0.859
	CFD/OFD	0.379 (0.211–0.546)	0.091	0.091	1.0
	HC/AC	0.138 (0.000–0.279)	−0.004	0	0.996
	Composite index	0.952 (0.868–1.000)	0.893	0.909	0.984

CPA, choroid plexus area; HA, head area; CPL, choroid plexus length; OFD, occipitofrontal diameter; CFD, the vertical distance from the apex of the CP to the medial side of the frontal bone; HC, head circumference; AC, abdominal circumference.

## Discussion

Unlike other non-lethal brain anomalies, such as posterior fossa malformations and corpus callosal anomalies, the fetuses included in our study were characterized by abnormal cerebrospinal fluid development. Based on the experience of our center and reports from previous studies, we found that the boundary of the lateral ventricle was unclear in the case group. Therefore, parameter measurements of the choroid plexus and head were obtained in the axial plane of the fetal head during first-trimester screening (5).

### Reference range in normal fetuses

To minimize the subjectivity of the measurements, we designed two new reference lines perpendicular to the falx cerebri through the apex of CPs to measure the CFD and CPL. CFD is a novel parameter that has not been measured in previous studies and may quantify the degree of choroid retrocontraction. Prasad et al. demonstrated that the CPA/HA and CPL/lateral ventricular length (VL) ratios were good predictors of severe VM with AUCs of 0.90 and 0.87, respectively (9). More importantly, we compared the CPL/OFD and CPA/HA ratios in normal fetuses for the first time, all of which were obtained at the level of the choroid plexus.

In the subsequent analysis, we investigated the correlation between each parameter and CRL. The results indicated that, as CRL increased, the CPA/HA and CPL/OFD ratio in normal fetuses showed linear decreases, while the CFD/OFD ratio increased, indicating the development of fetal CPs and the lateral ventricle. Based on this premise, the 5th and 95th percentile curves for each index were plotted. This provided a relatively objective foundation for identifying pathological cases, which had not been delineated in previous reports.

### Pathological cases

Manegold-Brauer et al. evaluated a sonographic approach to classify first-trimester VM associated with further sonographic anomalies (13). They found that the ratios between CP and lateral ventricle diameter (PDVDR), CP and lateral ventricle length (PLVLR), and CP and lateral ventricle area (PAVAR) were below the 5th percentile in 82.4, 94.1, and 94.1% of VM cases, respectively. Genetic examination of these fetuses revealed karyotype abnormality in 52.9% of cases. In view of the significant impact of cerebrospinal fluid circulation-related disorders on the lateral ventricle, we replaced the lateral ventricle parameters with head parameters, which have rarely been reported in previous studies. In fetuses with isolated VM in our cohort, the CPA/HA and CPL/OFD ratios were significantly decreased, whereas the CFD/OFD and HC/AC ratios were increased, consistent with the findings of Manegold-Brauer et al. Notably, our results intuitively indicated a significant increase in the CFD/OFD ratio in all fetuses with VM, suggesting that “CPs staying in a significant backward state” may be a crucial clue for isolating VM. ROC curve analysis further confirmed this novel observation, demonstrating that the combination of CFD/OFD with the HC/AC ratio provided superior diagnostic performance, with both sensitivity and specificity at higher levels.

Cerebral ventriculomegaly from mid-trimester is often observed in the fetus with OSB (14). On the contrary, the appearance of a “dry brain” in the first trimester can be easily visualized, which is associated with sustained reduced cerebrospinal fluid, downward movement of the cerebellum, and stenosis of the midbrain aqueduct (15). The diameter of the brainstem (BS) and BS–occipital bone distance (BSOB) were measured on the sagittal plane, and the BS/BSOB ratio was calculated to predict the presence of OSB (16, 17). Increased fetal choroid-plexus-to-head-size ratio is a new sign for the easy detection of OSB in the first trimester in recent studies (5, 18). In our findings, the combined CPA/HA, CPL/OFD, and CFD/OFD ratios objectively represented a reduction in cerebrospinal fluid and contraction of the lateral ventricle. The combination of the CPA/HA, CPL/OFD, and CFD/OFD ratios provided superior diagnostic performance, with a sensitivity of 94.1% and a specificity of 85.2%. Interestingly, the scatterplot indicates that the proportions of these three diagnostic criteria above the 95th centile were only 52.9, 35.3, and 52.9%, respectively. Therefore, it is evident that not all lateral ventricle areas are decreased in fetuses with OSB, particularly at early gestation. It is suggested that relying solely on subjective signs of “dry brain” may lead to a missed diagnosis of OSB.

Intrauterine infection, aneuploidy, and environmental exposure may serve as pathogenic factors for microcephaly (19, 20). Fetal microcephaly may constitute the initial phenotypic manifestation of genetic abnormalities or may be indicative of neurological malformation. The prognosis of microcephaly varies significantly depending on its underlying causes and severity (11). In our cohort, 90.9% of fetuses with microcephaly had a CPA/HA ratio > 95th centile, and 54.5% had a CPL/OFD ratio > 95th centile. Superior diagnostic performance was achieved by combining the CPA/HA, CPL/OFD ratios, with a sensitivity of 90.9% and a specificity of 98.4%, suggesting that timely evaluation using three-dimensional multiplanar imaging of cranial anatomy should be conducted after 16 weeks, when CPA/HA and CPL/OFD increase in the early stage. Furthermore, MRI, genetic testing, and intrauterine infection-related examinations would be warranted.

### Clinical implications

Ratios commonly used for the early evaluation of posterior fossa anomalies, such as the brainstem/occipital bone ratio (BS/OB), brainstem-to-occipital bone ratio (BS/BSOB), and intracranial translucency (IT), may be subject to potential confounding interpretations. These markers are known to vary with gestational age and crown–rump length (CRL) (21, 22). Furthermore, cutoff thresholds (e.g., 95th percentile) are not standardized across studies, and significant inter-study heterogeneity has been highlighted in systematic reviews (23). Therefore, although these ratios provide valuable diagnostic clues for early screening, they should be interpreted with caution.

Based on our findings, we recommend that the relevant parameters should be measured in doubtful cases during early pregnancy: (1) abnormal location of the CPs, such as staying in a significant “backward” or “suspension” state; (2) choroid-plexus-to-head disproportion, including the choroid plexus fills the entire lateral ventricle or the proportion is too small. Based on previous studies, we have for the first time quantitatively analyzed the state of the choroid plexus in the intracranial cavity of fetuses with nervous system abnormalities associated with abnormal cerebrospinal fluid development at early gestation. These ratios (e.g., CPA/HA, CPL/OFD, and CFD/OFD ratios) may also have potential for incorporation into AI-driven triage systems and structured reporting frameworks. Such applications could minimize inter-observer variability and promote consistency in early screening, although their clinical utility requires confirmation in larger, multicenter cohorts.

This study has several limitations that warrant consideration. First, the number of abnormal cases was relatively small, and some subcohorts included only a limited number of cases. This inevitably reduces statistical power and precludes robust subgroup conclusions, consistent with previous reports highlighting small series as a common challenge in prenatal imaging research (24, 25). Consequently, our subgroup findings should be regarded as exploratory and interpreted with caution. Another limitation is that it was conducted in a single-center setting with an ethnically homogenous cohort, which has been highlighted in previous studies (26). Therefore, the applicability of our results to more diverse populations should be interpreted with caution.

## Conclusion

Assessing choroid-plexus-to-head ratios in the first trimester appears promising for identifying nervous system malformations associated with abnormal cerebrospinal fluid development.

## Data Availability

The raw data supporting the conclusions of this article will be made available by the authors without undue reservation.

## Supplementary material

SUPPLEMENTARY FIGURE S1 Bland–Altman plots showing mean difference and 95% limits of agreement between paired measurements of the parameters in the choroid-plexus-to-head section by the same observer (intra-observer). CPA, choroid plexus area; HA, head area; CPL, choroid plexus length; OFD, occipitofrontal diameter; CFD, the vertical distance from the apex of the CP to the medial side of the frontal bone; HC, head circumference; AC, abdominal circumference.

SUPPLEMENTARY FIGURE S2 Bland–Altman plots showing mean difference and 95% limits of agreement between paired measurements of the parameters in the choroid-plexus-to-head section by two different observers (inter-observer). CPA, choroid plexus area; HA, head area; CPL, choroid plexus length; OFD, occipitofrontal diameter; CFD, the vertical distance from the apex of the CP to the medial side of the frontal bone; HC, head circumference; AC, abdominal circumference.

## Glossary

VM: Ventriculomegaly
OSB: Open spina bifida
CPA: Choroid plexus area
CPL: Choroid plexus length
OFD: Occipitofrontal diameter
HA: Head area
HC: Head circumference
AC: Abdominal circumference
CP: Choroid plexus
CFD: the vertical distance from the apex of the CP to the medial side of the frontal bone
CRL: Crown-rump length
SD: Standard deviation
MRI: Magnetic resonance imaging
NT: Nuchal translucency
IQR: Interquartile range
ICCs: Intraclass correlation coefficients
VL: Ventricular length
PDVDR: The ratio between CP and lateral ventricle diameter
PLVLR: The CP and lateral ventricle length
PAVAR: The CP and lateral ventricle area
BS: Brainstem
BSOB: BS–occipital bone distance
