# Supplementary figures and images for: First-trimester choroid plexus-to-head ratio: a novel sonographic marker for the early detection of fetal central nervous system malformations
Source: Front Med (Lausanne). 2025 Nov 5;12:1637954. doi: 10.3389/fmed.2025.1637954 (PMC12626977; doi:10.3389/fmed.2025.1637954)

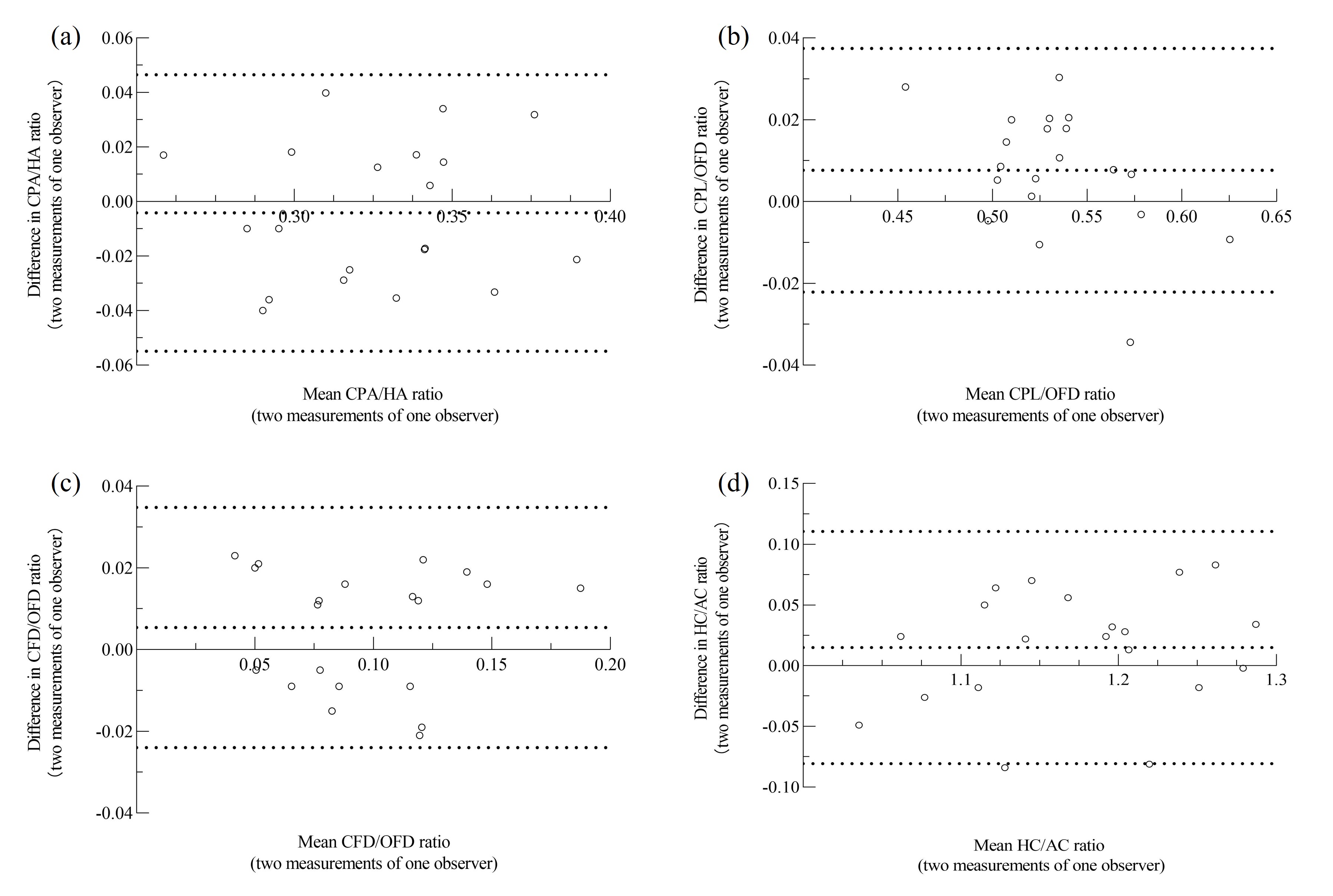

Supplement: SUPPLEMENTARY FIGURE S1 — Bland–Altman plots showing mean difference and 95% limits of agreement between paired measurements of the parameters in the choroid-plexus-to-head section by the same observer (intra-observer). CPA, choroid plexus area; HA, head area; CPL, choroid plexus length; OFD, occipitofrontal diameter; CFD, the vertical distance from the apex of the CP to the medial side of the frontal bone; HC, head circumference; AC, abdominal circumference. [file Image_1.JPEG]

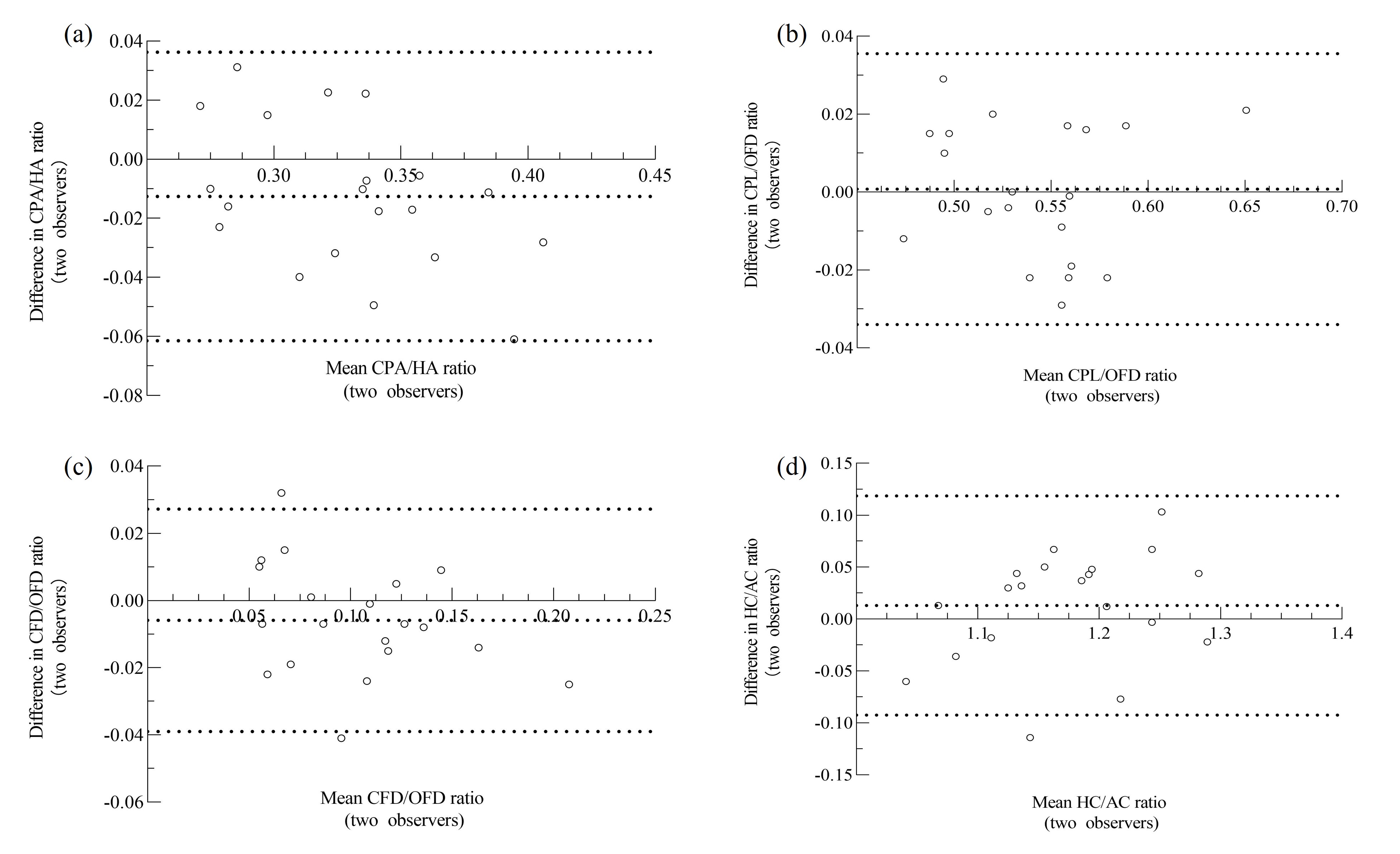

Supplement: SUPPLEMENTARY FIGURE S2 — Bland–Altman plots showing mean difference and 95% limits of agreement between paired measurements of the parameters in the choroid-plexus-to-head section by two different observers (inter-observer). CPA, choroid plexus area; HA, head area; CPL, choroid plexus length; OFD, occipitofrontal diameter; CFD, the vertical distance from the apex of the CP to the medial side of the frontal bone; HC, head circumference; AC, abdominal circumference. [file Image_2.JPEG]
